# Effects of 5α-dihydrotestosterone on the modulation of monocyte/macrophage response to *Staphylococcus aureus:* an in vitro study

**DOI:** 10.1186/s13293-023-00501-2

**Published:** 2023-03-31

**Authors:** Déborah Cruz Dos Santos, Rafaela de Souza Bittencout, Iago Dórea Arêas, Larissa Silva C. Pena, Carolline Florentino Almeida, Bruna Carolina de Brito Guimarães, Rafael Santos Dantas Miranda Dórea, Thiago Macêdo Lopes Correia, Manoel Neres Santos Júnior, Lorena Lôbo Brito Morbeck, Talita Costa Dos Santos, Clarissa Leal S. Souza, Samira Itana de Souza, Telma de Jesus Soares, Regiane Yatsuda, Guilherme Barreto Campos, Lucas Miranda Marques

**Affiliations:** 1 Multidisciplinary Institute of Health, Federal University of Bahia (UFBA), Rua Hormindo Barros, 58, Candeias, Vitória da Conquista, Bahia 45029-094 Brazil; 2 University of Santa Cruz (UESC), Campus Soane Nazaré de Andrade, Ilhéus, Brazil; 3 Department of Microbiology, Institute of Biomedical Science, University of São Paulo, São Paulo, Brazil; 4 Federal University of Bahia (UFBA), Salvador, Brazil

**Keywords:** 5α-Dihydrotestosterone, *Staphylococcus aureus*, Inflammatory response

## Abstract

**Background:**

*Staphylococcus aureus* (*S. aureus*) is a pathogen responsible for a wide range of clinical manifestations and potentially fatal conditions. There is a paucity of information on the influence of androgens in the immune response to *S. aureus* infection. In this study, we evaluated the influence of the hormone 5α-dihydrotestosterone (DHT) on mouse peritoneal macrophages (MPMs) and human peripheral blood monocytes (HPBMs) induced by *S. aureus*.

**Methods:**

An in vitro model of MPMs from BALB/c sham males, orchiectomised (OQX) males, and females was used. Cells were inoculated with 10 μL of *S. aureus*, phage-type 80 or sterile saline (control) for 6 h. The MPMs of OQX males and females were pre-treated with 100 μL of 10^–2^ M DHT for 24 h before inoculation with *S. aureus*. The concentration of the cytokines TNF-α, IL-1α, IL-6, IL-8, and IL-10; total nitrites (NO^−2^); and hydrogen peroxide (H_2_O_2_) were measured in the supernatant of MPM cultures. In addition, the toll-like receptor 2 (*TLR2*) and nuclear factor kappa B (*NF-kB*) genes that are involved in immune responses were analysed. For the in vitro model of HPBMs, nine men and nine women of childbearing age were selected and HPBMs were isolated from samples of the volunteers’ peripheral blood. In women, blood was collected during the periovulatory period. The HPBMs were inoculated with *S. aureus* for 6 h and the supernatant was collected for the analysis of cytokines TNF-α, IL-6, IL-12; and GM-CSF, NO^−2^, and H_2_O_2_. The HPBMs were then removed for the analysis of 84 genes involved in the host’s response to bacterial infections by RT-PCR array. GraphPad was used for statistical analysis with a *p* value < 0.05.

**Results:**

Our data demonstrated that MPMs from sham males inoculated with *S. aureus* displayed higher concentrations of inflammatory cytokines and lower concentrations of IL-10, NO^−2^, and H_2_O_2_ when compared with MPMs from OQX males and females. A similar result was observed in the HPBMs of men when compared with those of women. Previous treatment with DHT in women HPBMs increased the production of pro-inflammatory cytokines and decreased the levels of IL-10, NO^−2^, and H_2_O_2_. The analysis of gene expression showed that DHT increased the activity of the TLR2 and NF-kB pathways in both MPMs and HPBMs.

**Conclusions:**

We found that DHT acts as an inflammatory modulator in the monocyte/macrophage response induced by *S. aureus* and females exhibit a better immune defence response against this pathogen.

## Highlights

- A sex difference in the immune response to *S. aureus* was observed.
- DHT stimulated a greater release of inflammatory cytokines.
- DHT promoted the activation of genes involved in this response.
- DHT suppressed the release of inflammatory markers.

## Introduction

*Staphylococcus aureus* (*S. aureus)* is a pathogen responsible for a variety of clinical manifestations ranging from relatively benign skin infections to potentially life-threatening conditions, such as endocarditis and osteomyelitis. *S. aureus* is also a commensal bacterium that colonises approximately 30% of the human population, with a higher risk of complications and mortality in men [1–3]. According to some studies, the sex of an individual serves as a contributory factor in the incidence and progression of disorders associated with the immune response. Accumulated evidence shows that sex can also play an important role in susceptibility to infectious diseases. In general, females generate more robust humoral and cell-mediated immune responses after exposure to an antigen when compared with males. Furthermore, males frequently mount heightened inflammatory immune responses to microbial stimuli, thereby worsening disease prognosis [4, 5].

In general, while oestrogen has an immune-stimulating effect, testosterone has an immunosuppressive effect, thereby increasing susceptibility to infection and colonisation by *S. aureus* [6–9]. Men are more susceptible to sepsis, acute respiratory distress, and multiple organ failure following soft tissue traumatic haemorrhagic shock and thermal injury, in part as a result of immune suppression and abnormal neutrophil activation [10]. A correlation exists between the parasitic load, strength of immune responses, and levels of sex hormones in men, and it has been observed that both low parasitic loads and weakened immune responses are caused by the immunosuppressive effects of testosterone on immune cells [11].

Women also display reduced infection rates for a variety of bacteria, viruses, and parasites, such as *Helicobacter pylori* [12], *Mycobacterium tuberculosis* [13], hepatitis B virus [14], and *Aspergillus fumigatus* [5]. In addition, an analysis of COVID-19 virus-related deaths among 17 million adults showed that females were more protected against severe COVID-19 infection and mortality. Males have a 1.59 times greater risk ratio of developing virus-related complications than females [15]. However, the molecular mechanisms underlying sexually dimorphic immune responses are not fully understood.

Sex hormones such as oestrogen and testosterone have been reported to modulate the differentiation, maturation, lifespan, and effector functions of innate immune cells, including neutrophils, macrophages, natural killer (NK) cells, and dendritic cells [5]. Macrophages perform several functions in the immune system. They facilitate the elimination of invading pathogens by phagocytosis, production of reactive oxygen/nitrogen species, and release of several inflammatory mediators, such as cytokines. These cytokines are fundamental to the initiation and propagation of the inflammatory process to contain pathogens and promote homeostatic regulation [4, 16]. There are few studies regarding the role of testosterone in *S. aureus* infection of macrophages.

To better understand the role of androgens in the immune response against *S. aureus*, this study assessed the effects of 5α-dihydrotestosterone (DHT) on the profile of cytokines, inflammatory mediators as NO^−2^ and H_2_O_2_, genes associated with this response to *S. aureus* mediated by murine peritoneal macrophages (MPMs), and human peripheral blood monocytes (HPBMs). There is limited information concerning the role of DHT in the response to infections caused by *S. aureus*. Despite the impressive advances in many medical fields, the pathophysiology, diagnostic procedures, and appropriate therapeutic interventions for *S. aureus* infections are still a matter of debate [17–19]. The model stimulated by *S. aureus* developed in this study was based on the studies cited.

## Materials and methods

### Microorganisms

The phage-type used in this study was *Staphylococcus aureus* 80 from the microbiology laboratory of the Federal University of Bahia, Campos Anísio Teixeira, Vitória da Conquista-Ba. This is the same strain used in previous studies [20, 21] and is derived from the nasal swabs of children attending day care centres in the municipality. Brain heart infusion (BHI) and mannitol salt agar were used to activate, culture, and subculture *S. aureus* strains for subsequent inoculation of MPMs and HPBMs. In addition, Gram staining, catalase and coagulase tests, and PCR (nuc gene) were performed to confirm the identity and purity of the bacterial strain. The inoculum of *S. aureus* was obtained by direct suspension, removing 3–5 colonies (same morphological type) from the plates containing the reference strain. The colonies were then transferred to tubes containing sterile saline solution, vortexed, and analysed using a spectrophotometer to obtain the following parameters: absorbance of 0.135 at 660 nm that is equivalent to 1 × 10^8^ colony forming units (CFU) [22]**.**

### In vitro model of MPMs

#### Animals

Six male and three female specific pathogen-free (SPF) Balb/c mice aged between 6 and 8 weeks were used. The mice used in the in vitro experiments were obtained from the Animal Laboratory of the Multidisciplinary Centre for Biological Research of the State University of Campinas (CEMIB/UNICAMP-SP) and kept under controlled conditions of light (light from 7 am to 7 pm) and temperature (23 ± 3 °C), with free access to water and food in the animal facility of the Multidisciplinary Institute of Health at the Federal University of Bahia (IMS/UFBA).

#### Orchiectomy and sham surgery

The animals underwent surgery to remove the gonads (orchiectomy) to significantly decrease their levels of sex hormones. Following administration of anaesthesia, the orchiectomy or simulated surgery was performed using a trans scrotal approach. A 1-cm midline scrotal incision was made, through which the testes and epididymis were exposed and removed. The incision was then sutured after the resection of both testes. For sham mice, the testes were visualised but not removed [24]. The sham surgery was performed as the control [25]. The animals were anesthetised with ketamine and xylazine at doses of 5 and 50 mg/kg, respectively. After surgery, the animals received prophylactic treatment with 2.5% of the antibiotic enrofloxacin in a single dose of 2.5 mg/kg [22]. OQX animals remained under observation for 14 days to confirm the reduction of testosterone levels from the gonads using enzyme-linked immunosorbent assay (ELISA) (Cayman Chemical’s ACE™, Ann Arbor, MI, USA). After this period, macrophage recruitment was induced in the peritoneal cavity of OQX and sham males.

#### Females

The oestrous cycles of females were monitored by vaginal lavage, which was visualised on a slide under an optical microscope. When females were in diestrum, intraperitoneal macrophages were stimulated and proestrum, the phase of the cycle with the highest levels of 17b-estradiol (E2), was confirmed after 72 h. No surgery was performed (ovariectomy) in females. Females were used to assess the effect of sex on the immune response of HPBMs to *S. aureus*.

#### Isolation of male and female MPMs

To induce macrophages recruitment into the peritoneal cavity, 2 mL of thioglycolate medium (3%) was injected into the peritoneal cavity of OQX (*n* = 3), sham (*n* = 3), and female (*n* = 3) mice for increasing monocyte migration into the peritoneum, thereby increasing macrophages yield. Peritoneal macrophages are more mature after isolation and more stable in their functions and phenotype [23]. The animals were euthanized after 3 days by decapitation with the aid of a guillotine. Cells were isolated from the peritoneal lavage and cultured in RPMI 1640 medium (Gibco™) supplemented with 10% foetal bovine serum (FBS) (Sigma Aldrich) and ciprofloxacin (20 µg/mL) (Sigma Aldrich). The MPM cell suspension (2 × 10^5^/mL) was seeded in 24-well plates [24].

#### Inoculation of MPMs with *S. aureus* or sterile saline solution and treatment with DHT

MPMs from male sham, male OQX, and female mice were cultured with 10 μL of the inoculum of *S. aureus* (10^8^ CFU) for 6 h at 37 °C in 5% CO_2_ and 95% humidified air [24]. Part of the cells were pre-treated with 100 μL of 10^–2^ M DHT for 24 h at 37 °C before inoculation with *S. aureus* or addition of 10 μL of sterile saline solution. Male and female MPMs were plated as follows: cells with saline solution (control) (6 h); Cells with *S. aureus* (6 h); Cells pre-treated with DHT (24 h) and then infected with *S. aureus* (6 h). DHT was used in place of testosterone, since it is the active androgen that is not converted to E2 [25]. The DHT concentration used was determined by following the manufacturer's instructions (Sigma Aldrich Brazil LTDA-5α-Dihydrotestosterone-D3 (16, 16, 17-D3) Solution). For dose selection, we conducted a pilot study based on different doses (DHT 10^–1^ M–10^–9^ M—data not shown) and the dose of choice was 10^–2^ M. The incubation time was similar to that of other studies using macrophages cell cultures treated in vitro with testosterone [26, 27].

#### Dosage of cytokines

The levels of cytokines TNF-α, IL-1α, IL-6, IL-8, and IL-10 were measured in the supernatant of MPM cell cultures by ELISA using the commercial ProcartaPlex™ Simplex Kit Invitrogen (Thermo Fisher, São Paulo, Brazil). The assay was performed following the manufacturer’s instructions and the cytokine levels are expressed in absolute values (pg/mL). All groups were analysed in triplicate.

#### Gene expression analysis of receptors and transcription factors by RT-qPCR

Cells were detached from the wells using trypsin and stored at -80 °C for gene expression analysis. The relative gene expressions of *TLR2* and *NF-κB* were determined. Gene expression was analysed by RT-qPCR arrays. The gene expression assay was performed on plates with the *NF-κB* and *TLR2* target genes using the SYBR^®^ PCR Master Mix (Applied Biosystems™). Amplification was performed using the StepOnePlus thermal cycler with the following cycles: 50 °C for 10 min, 95 °C for 10 min, 45 cycles of 95 °C for 15 s, and 60 °C for 1 min. Data were analysed using the comparative method (2^ΔΔCt^) and normalised based on GAPDH gene expression according to the manufacturer’s instructions.

### In vitro model of HPBMs

#### Selection of study participants

Peripheral blood samples were obtained from men and women aged 18–25 years, who volunteered to be included in the study. Volunteers were screened using questionnaires, which were provided. Information on the participants’ date of birth, sex, clinical history, and lifestyle habits, such as alcohol use or smoking, and medication use, was collected. The inclusion criteria were: (1) healthy, young volunteers who had not used antibiotics, antifungals, or anti-inflammatory drugs in the last 90 days and (2) women of childbearing age with a regular menstrual cycle in the fertile period of their menstrual cycle. The exclusion criteria were: (1) women who used hormonal contraceptives, (2) breastfeeding, pregnant, and puerperal women, (3) volunteers who consume alcohol or use tobacco, (4) volunteers who had performed physical activity 24 h prior to blood collection, and (5) volunteers with human immunodeficiency virus (HIV) infection or other infections and immune inflammatory diseases including diabetes, cardiopathies, psychiatric diseases, cancer, and kidney disease, and volunteers presenting with clinical signs and symptoms, such as fever, sore throat, skin rash, allergy, or flu. This study was approved by the Ethics Committee in Research on Human Beings (CEP) of the same institution (CAAE:95158218.3.0000.5556). The study met ethical requirements by complying with the guidelines of Resolution 466/12.

#### Sampling procedure

Phlebotomies for the collection of peripheral blood samples were performed by qualified professionals using sterile materials and personal protective equipment (PPE). All blood samples were kept at 6–10 °C, and monocytes were isolated within 2 h of blood collection. A portion of the collected blood was used to determine the levels of hormones, including testosterone, E2, progesterone, follicle-stimulating hormone (FSH), and luteinizing hormone (LH). Furthermore, the general health status of the individual was evaluated by measuring glucose, total serum cholesterol, glutamic–oxaloacetic transaminase (GOT), glutamic pyruvic transaminase (GPT), and complete blood count. For women, sample collection dates were determined based on their menstrual cycle.

#### Isolation of HPBMs

Peripheral blood mononuclear cells (PBMC) were extracted from the blood using Ficoll, a hydrophilic polysaccharide density gradient medium to separate the blood into its components. Once PBMCs were isolated, it was possible to separate the monocytes from lymphocytes using techniques based on different properties of cells, such as their unique size and density compared to lymphocytes, and their ability to adhere to glass or plastic. Owing to the risk of lymphocyte contamination, Percoll, a hyperosmolar percolator gradient (density = 1064 g/mL), was used. Peripheral blood was mixed with PBS in a 1:1 ratio. PBMCs were separated by Ficoll column centrifugation (400 × *g* for 20 min), washed, and centrifuged twice with 1 × PBS at 100 × *g* for 10 min. Subsequently, the mononuclear cells were separated by Percoll column centrifugation at 400 × *g* for 35 min and resuspended in RPMI 1640 medium containing 10% foetal bovine serum, 2 mM glutamine, 10 mM HEPES, and 20 μl/mL ciprofloxacin. After the evaluation of viability and adequate confluence (2 × 10^5^/mL), the cells were plated in 24-well polystyrene plates and incubated for 24 h at 37 °C with 5% CO_2_ before inoculation with *S. aureus* [28]. This procedure allowed us to obtain a homogeneous population of HPBMs, which appeared as adherent cells [24].

#### Inoculation of HPBMs with *S. aureus* or sterile saline solution and pre-treatment with DHT

HPBMs were grown using 10 μl of *S. aureus* inoculum (CFU 10^8^) for 6 h in 5% CO_2_ and 95% humidified air at 37 °C [24]. Additional cells in triplicate were pre-treated with 100 μl DHT (10^–2^ M) for 24 h at 37 °C before stimulation with *S. aureus* or 10 μl of sterile saline solution. The HPBMs were divided into plaques: cells with *S. aureus* (6 h), cells with sterile saline solution (control) (6 h), and cells pre-treated with DHT (24 h), and subsequently infected with *S. aureus* (6 h).

#### Cytokine concentrations of HPBMs

To determine cytokine concentrations, the cell culture supernatants were obtained from the culture plate wells and stored at – 80 °C. The concentrations of TNF-α, IL-12 and GM-CSF were determined using Luminex xMAP^®^ with the Procartaplex Immunoassay kit (Affymetrix eBioscience) according to the manufacturer’s instructions.

#### Gene expression analysis of HPBMs

With the aid of trypsin, the HPBMs were removed from the cell culture wells and stored in RNAlater at − 80 °C for gene expression. The expression of inflammatory markers was evaluated using quantitative reverse transcription PCR (RT-qPCR). The mRNA was extracted using TRIzol^®^ LS (Life Technologies™) following the manufacturer’s protocol. The cDNA was obtained from mRNA by retro-transcription (RT), using the SuperScript^®^ III Reverse Transcriptase kit with additional complementary oligonucleotides to the mRNA poly-A tail (Oligo Dt) and RNAse inhibitor. The obtained cDNA was subjected to analysis using the Human Innate and Adaptive Immune Responses PCR Array (Qiagen-SABioscience) for the evaluation of genes involved in the response of the host’s monocytes to bacterial infection. The qPCR array kit was used to analyse the expression of 84 genes involved in the host response to bacterial infection, including genes related to TLR signalling, and genes involved in the acute phase response, complement system activation, inflammatory response, and acquired immune response against bacteria. All procedures and data analyses were performed in accordance with the manufacturer’s instructions and software.

### Inflammatory markers

#### Measurement of NO^−2^ concentration in MPMs and HPBMs

Nitric oxide (NO) levels were quantified using the Griess assay. Briefly, 50 µL aliquots of the test solution (standards and experimental samples) were added to 0.1% Griess reagent solution (50 µL of 3.9 mM N-(1-naphthyl)ethylenediamine in 5% (v/v) phosphoric acid) and incubated in the dark at room temperature for 10 min. Sulphanilamide solution (1% in phosphoric acid) was added to the mixture, and after 10 min of incubation the absorbance of the coloured product was measured at 540 nm using a microplate reader. Standard sodium nitrate solutions (1.65–100 mM) were used to construct a standard curve to determine the concentrations of the samples [29]. Since nitric oxide is a difficult gas to detect, the concentration of nitrites was determined by indirect measurement of the levels of total nitrites by this reaction in a 96-well plate.

#### Measurement of H_2_O_2_ concentration in MPMs and HPBMs

For determining H_2_O_2_ concentration, an aliquot of each cell culture supernatant was added to a 96-well plate, in triplicates, using the Amplex^®^ Red Hydrogen Peroxide/Peroxidase (Invitrogen) kit, according to the manufacturers’ instructions. The absorbance was measured at 560 nm using a spectrophotometer [30].

### Statistical analysis

Statistical analyses were performed using the *Graphpad-Prism 5.0* (*Graphpad Software, San Diego, CA-USA*). The results were expressed as mean ± standard deviation (SD). The *Shapiro Wilk* test was used to evaluate data normality. To determine statistical differences in values between two different groups with normal distribution and variance homogeneity the *Student’s T test* was used; otherwise, the *Mann Whitney* test was used. Statistical differences were considered significant at *p* < 0.05, using a 95% confidence interval.

## Results

### In vitro model of MPMs

#### MPMs from sham males express more inflammatory cytokines (TNF-α, IL-1α, IL-8) than those from OQX males, while those from females express more IL-10

**Fig. 1:**
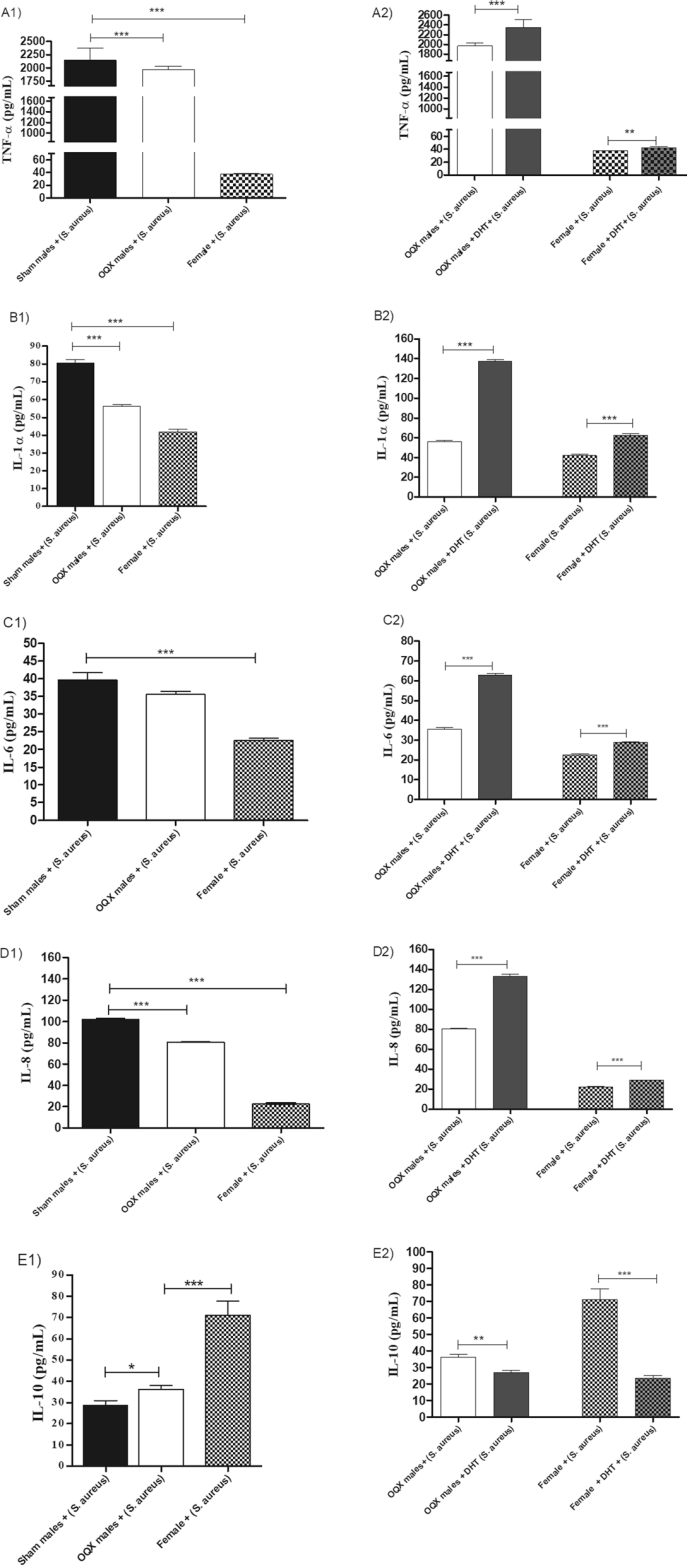
TNF-α (**A1**), IL-1α (**B1**), IL-6 (**C1**), IL-8 (**D1**), IL-10 (**E1**) concentrations in the supernatant of MPMs from sham males, OQX males and females. MPMs inoculated with 10 µL of *S. aureus* [6 h (10^8^ CFU)]. TNF-α (**A2**), IL-1α (**B2**), IL-6 (**C2**), IL-8 (**D2**), IL-10 (**E2**) concentrations in the supernatant of MPMs from sham males, OQX males and females: pre-treated with 100 µL of DHT [24 h (10^–2^ M)] and inoculated with 10 µL of *S. aureus* [6 h (10^8^ CFU)]. Data are expressed as mean ± SDM. *p < 0.05, **p < 0.01, ***p < 0.001

MPMs from sham males inoculated with *S. aureus* exhibited higher concentrations of inflammatory cytokines TNF-α, (Fig. 1A1) IL-1α (Fig. 1B1), IL-6 (Fig. 1C1), and IL-8 (Fig. 1D1) when compared with MPMs from females. However, those of females expressed higher IL-10 (Fig. 1E1).

#### DHT increases the expression of inflammatory cytokines and decreases levels of IL-10 in MPMs of OQX males and females

MPMs from OQX males and females inoculated with *S. aureus* and pre-treated with DHT exhibited a significant increase in the production of inflammatory cytokines TNF-α (Fig. 1A2), IL-1α (Fig. 1B2), IL-6 (Fig. 1C2), and IL-8 (Fig. 1D2) when compared with untreated MPMs from OQX males and females. However, DHT decreased IL-10 levels in MPMs from OQX males and females (Fig. 1E2) inoculated with *S. aureus*.

#### Concentration of NO^−2^ and H_2_O_2_ in MPMs from sham males, OQX males, and females

**Fig. 2:**
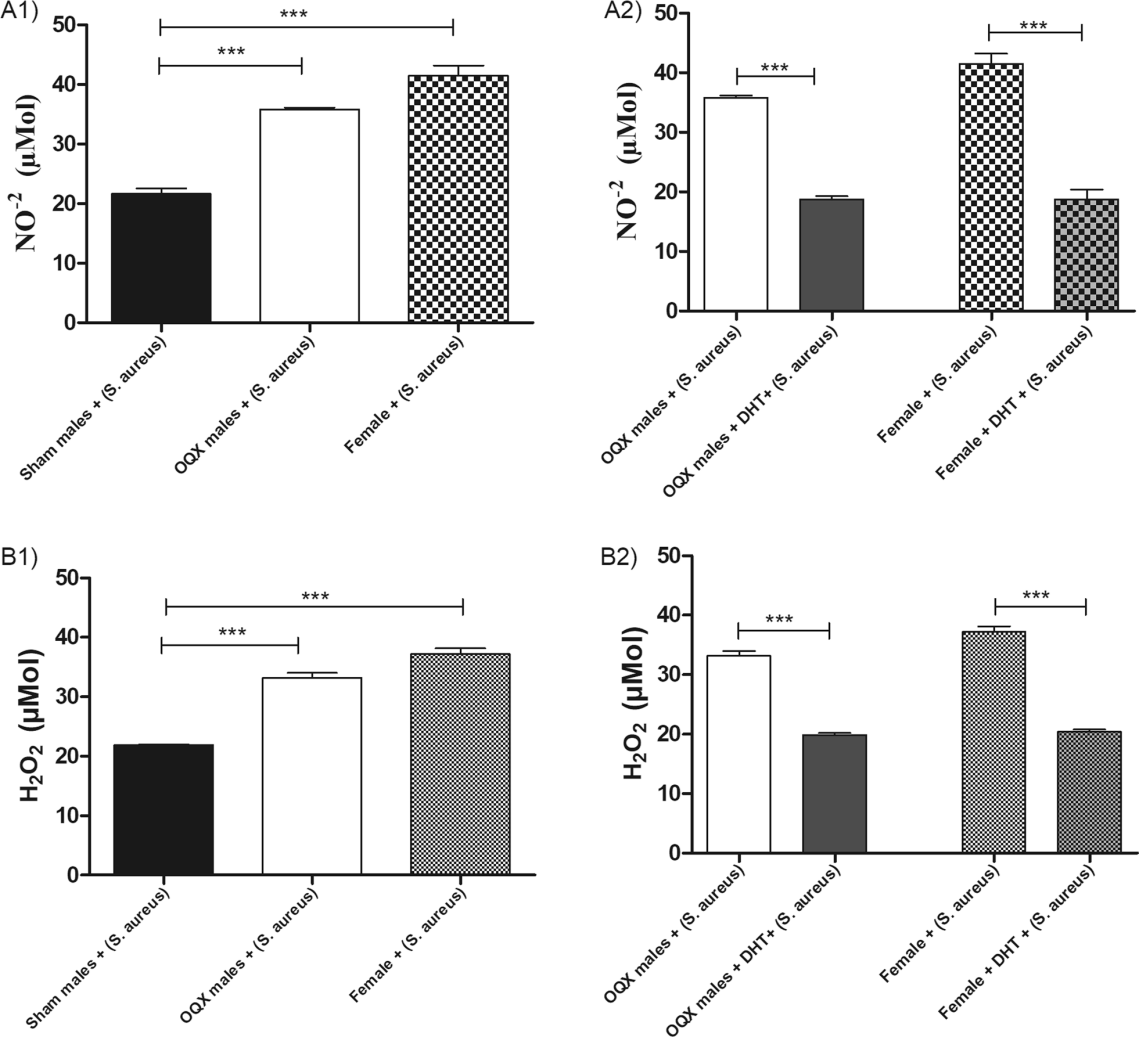
Total nitrite- NO^−2^ (**A**) concentration in the supernatant of MPMs from sham males, OQX males, and females. (**A1**): Sham males, OQX males and females: inoculated with 10 µL of *S. aureus* [6 h (10^8^ CFU)]. **A2** Sham males, OQX males, and females: pre-treated with 100 µL of DHT [24 h (10^–2^ M)] and inoculated with 10 µL of *S. aureus* [6 h (10^8^ CFU)]. H_2_O_2_ (**B**) concentration in the supernatant of MPMs from sham males, OQX males, and females. **B1** Sham males, OQX males, and females: inoculated with 10 µL of *S. aureus* [6 h (10^8^ CFU)]. **B2** Sham males, OQX males, and females: pre-treated with 100 µL of DHT [24 h (10^–2^ M)] and inoculated with 10 µL of *S. aureus* [6 h (10^8^ CFU)]. Data are expressed as mean ± SDM. *p < 0.05, **p < 0.01, ***p < 0.001

MPMs from sham males inoculated with *S. aureus* exhibited lower concentrations of NO^−2^ (Fig. 2A1) and H_2_O_2_ (Fig. 2B1) when compared with MPMs from OQX males and females. MPMs from OQX males and females inoculated with *S. aureus* and pre-treated with DHT exhibited a significant reduction in NO^−2^ (Fig. 2A2) and H_2_O_2_ (Fig. 2B2) production when compared with untreated MPMs from OQX males and females.

#### Relative gene expressions of TLR2 and NF-κB in MPMs from sham males, OQX males, and females inoculated with *S. aureus* and pre-treated with DHT

**Fig. 3:**
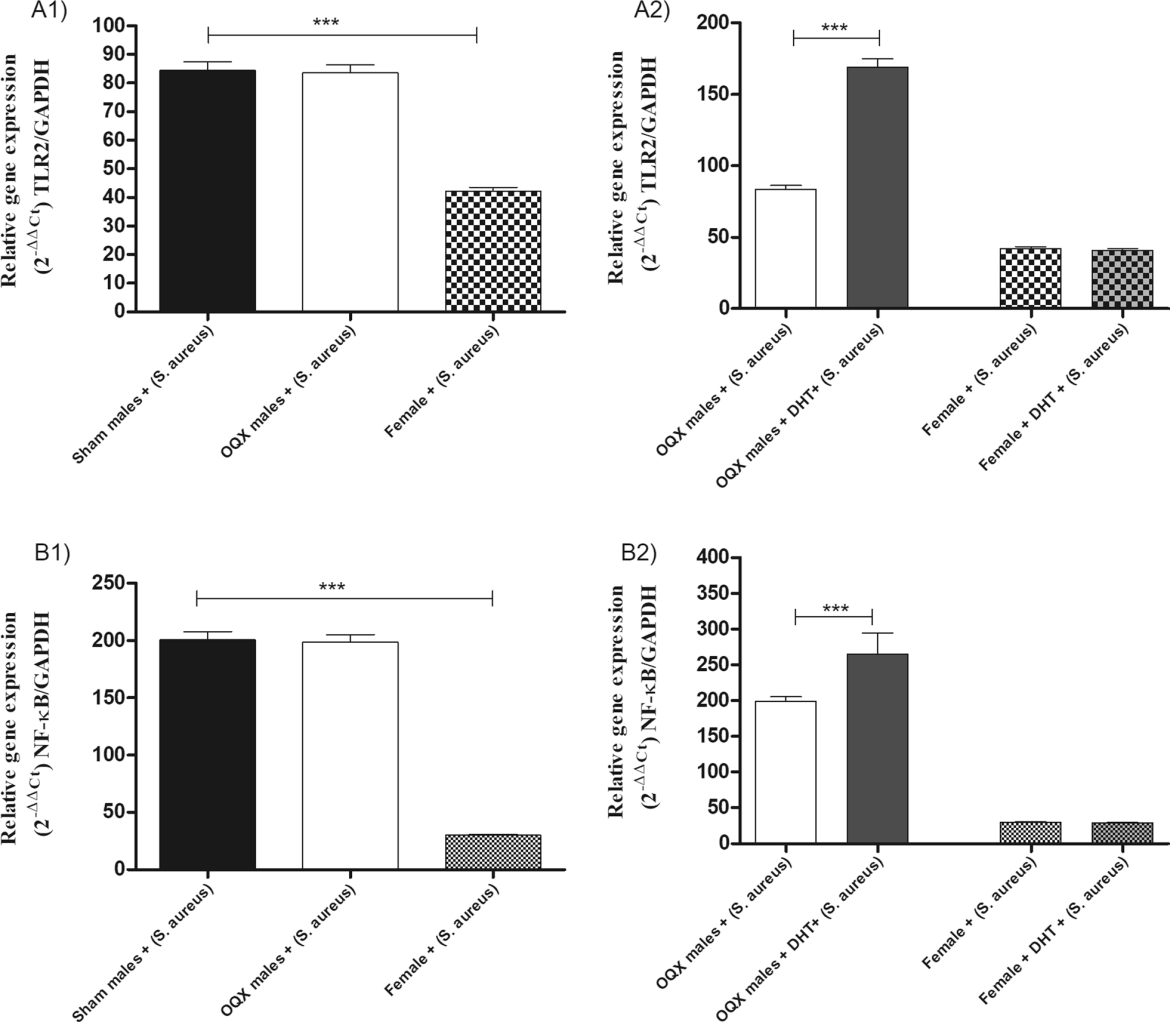
Relative expression of TLR2 (**A**) of MPMs from sham males, OQX males, and females. **A1** Sham males, OQX males, and females: inoculated with 10 µL of *S. aureus* [6 h (10^8^ CFU)]; **A2** Sham males, OQX males, and females: pre-treated with 100 µL of DHT [24 h (10^–2^ M)] and inoculated with 10 µL of *S. aureus* [6 h (10^8^ CFU)]. Relative expression of NF-κB (**B**) of MPMs from sham males, OQX males, and females. **B1** Sham males, OQX males, and females: inoculated with 10 µL of *S. aureus* [6 h (10^8^ CFU)]; **(B2**): Sham males, OQX males, and females: pre-treated with 100 µL of DHT [24 h (10^–2^ M)] and inoculated with 10 µL of *S. aureus* [6 h (10^8^ CFU)]. Data are expressed as mean ± SDM. *p < 0.05, **p < 0.01, ***p < 0.001

No differences were observed in TLR2 and NF-κB expression between MPMs from sham and OQX males. However, MPMs from sham males exhibited higher TLR2 (Fig. 3A1) and NF-κB (Fig. 3B1) expression levels when compared with MPMs from females. MPMs from OQX males pre-treated with DHT exhibited a significant increase in TLR2 (Fig. 3A2) and NF-κB (Fig. 3B2) expression. However, no significant differences were observed in expression of these genes in MPMs from females pre-treated with DHT.

### In vitro model of HPBMs

#### HPBMs from men express more inflammatory cytokines than HPBMs of women, while those from women express more IL-10

**Fig. 4:**
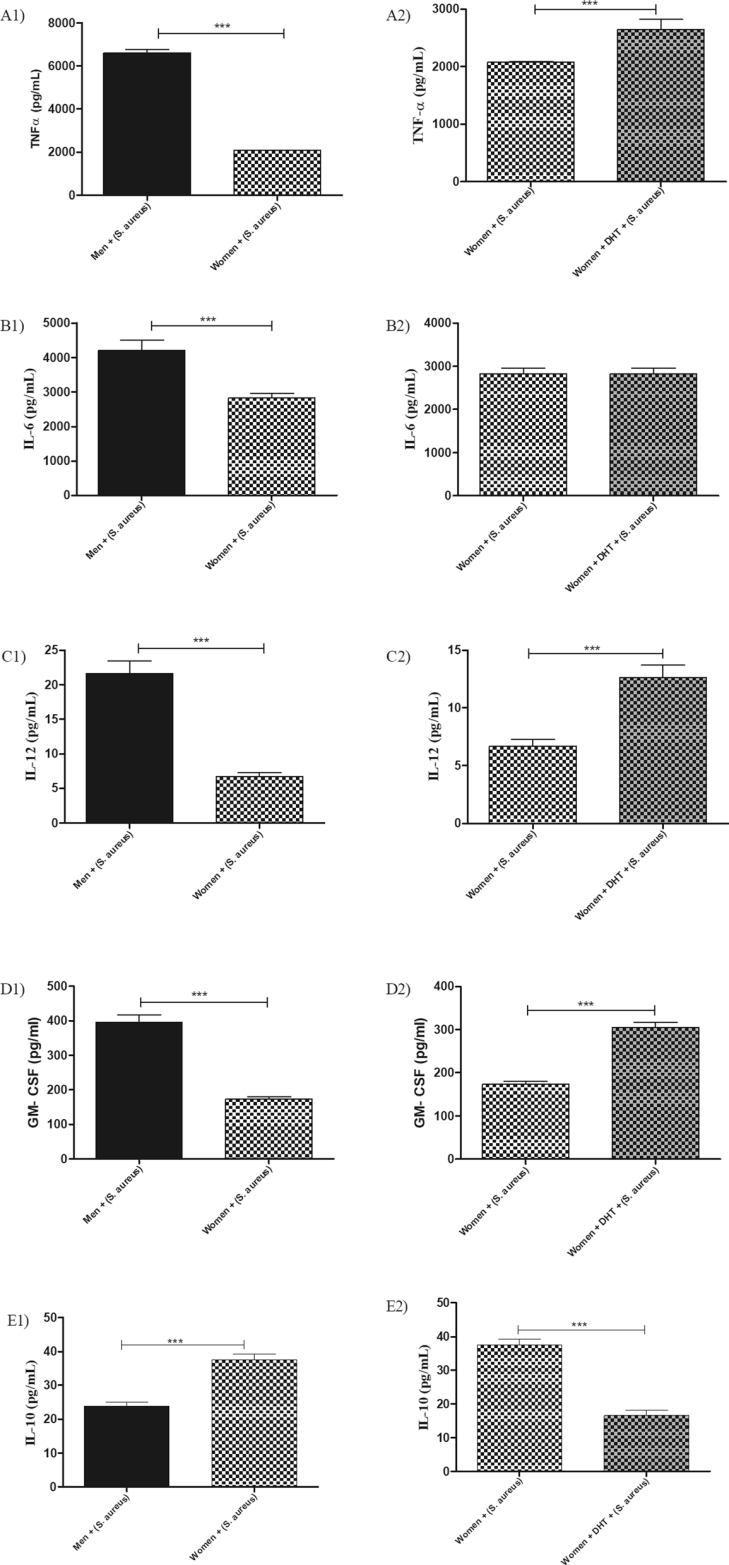
TNF-α (**A1**), IL-6 (**B1**), IL-10 (**C1**), IL-12 (**D1**), GM-CSF (**E1**) concentrations in the supernatant of HPBMs from men and women. HPBMs inoculated with 10 µL of *S. aureus* [6 h (10^8^ CFU)]. TNF-α (**A2**), IL-6 (**B2**), IL-10 (**C2**), IL-12 (**D2**), GM-CSF (**E2**) concentrations in the supernatant of HPBMs from men and women: pre-treated with 100 µL of DHT [24 h (10^–2^ M)] and inoculated with 10 µL of *S. aureus* [6 h (10^8^ CFU)]. Data are expressed as mean ± SDM. *p < 0.05, **p < 0.01, ***p < 0.001

The secretion of TNF-α (Fig. 4A1), IL-6 (Fig. 4B1), IL-12 (Fig. 4C1), and GM-CSF (Fig. 4D1) in HPBMs of men was significantly higher than that in women. However, the secretion of IL-10 (Fig. 4E1) in HPBMs of men was significantly lower than that in women.

#### DHT increases the secretion of cytokines TNF-α, IL-12, and GM-CSF in HPBMs of women

The HPBMs of women pre-treated with DHT exhibited a significant increase in the secretion of TNF-α (Fig. 4A2), IL-12 (Fig. 4C2), and GM-CSF (Fig. 4D2) when compared with the HPBMs of women. However, the HPBMs of women pre-treated with DHT exhibited a significantly reduced secretion of IL-10 when compared with the HPBMs of women (Fig. 4E2).

#### Total NO^−2^ and H_2_O_2_ concentrations in HPBMs

**Fig. 5:**
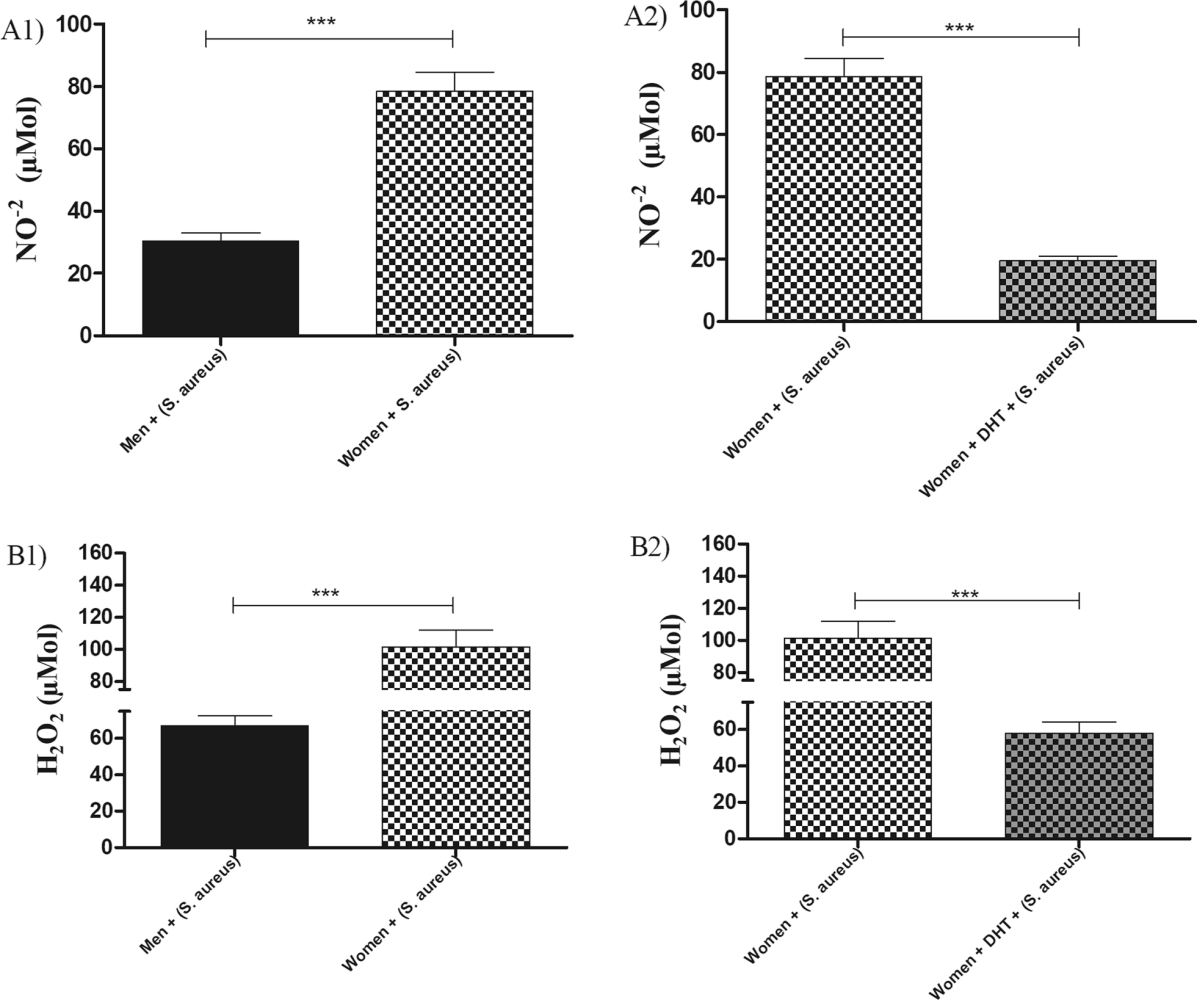
Total nitrite (**A**) concentration in the supernatant of HPBMs from men and women. **A1** HPBMs inoculated with 10 µL of *S. aureus* [6 h (10^8^ CFU)]. **A2** HPBMs pre-treated with 100 µL of DHT [24 h (10^–2^ M)] and inoculated with 10 µL of *S. aureus* [6 h (10^8^ CFU)]. H_2_O_2_ (**B**) concentration in the supernatant of HPBMs from men and women. **B1** HPBMs inoculated with 10 µL of *S. aureus* [6 h (10^8^ CFU)]. **B2** HPBMs pre-treated with 100 µL of DHT [24 h (10^–2^ M)] and inoculated with 10 µL of *S. aureus* [6 h (10^8^ CFU)]. Data are expressed as mean ± SDM. *p < 0.05, **p < 0.01, ***p < 0.001

The HPBMs of men produced significantly less NO^−2^ (Fig. 5A1) and H_2_O_2_ (Fig. 5B1) than women. Reduced production of NO^−2^ (Fig. 5A2) and H_2_O_2_ (Fig. 5B2) was observed in HPBMs of women pre-treated with DHT when compared with women.

#### Comparison of the gene expression of HPBMs of men and women inoculated with *S. aureus* or sterile saline

**Fig. 6:**
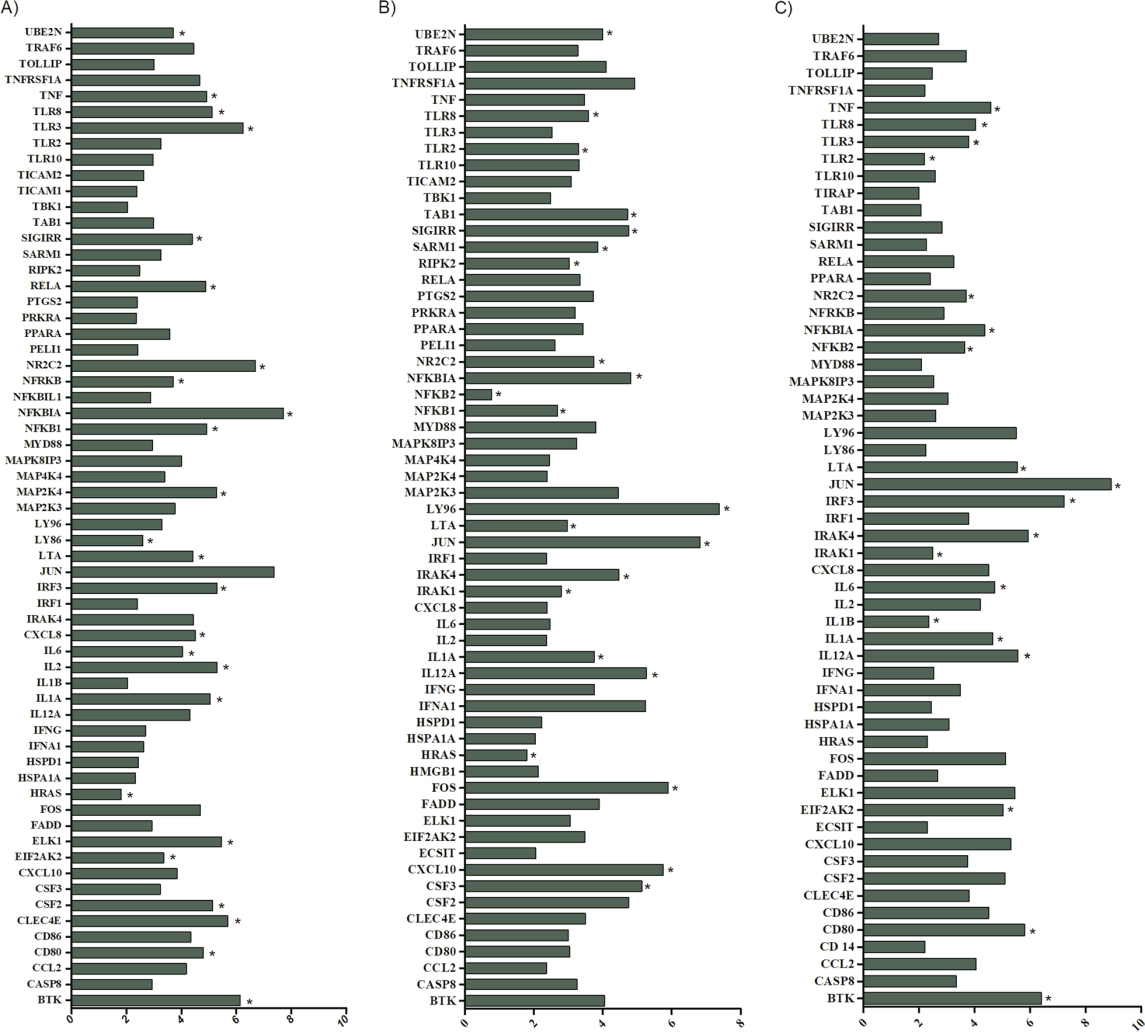
Analysis of 84 genes involved in the host response to bacterial infections. **A** HPBMs inoculated with *S. aureus* when compared to HPBMs inoculated with sterile saline of men and women. **B** HPBMs inoculated with *S. aureus* from men when compared to HPBMs from women. **C** HPBMs inoculated with *S. aureus* from DHT-treated women compared to HPBMs from Women. Analysis performed with the Human Innate and Adaptive Immune Responses PCR Array kit (Qiagen—SABioscience). Upregulated genes (green). Data are expressed as mean ± DPM. *p < 0.05 (non-parametric Mann–Whitney one-tailed test)

Among the 84 genes analysed, a statistically significant upregulation of 25 genes, i.e.,* BTK, CD80, CLEC4E, CSF2, EIF2AK2, ELK1, HRAS, IL1A, IL2, IL6, CXCL8, IRF3, LTA, LY86, MAP2K4, NKKB1, NFKBIA, NFRKB, NR2C2, RELA, SIGIRR, TLR3, TLR8, TNF,* and *UBE2N,* was observed HPBMs from men and women inoculated with *S. aureus* when compared to HPBMs inoculated with sterile saline (*p* < 0.05) (Fig. 6A).

#### Comparison of the gene expressions of HPBMs of men and women

Among the 84 genes analysed, a statistically significant upregulation of 22 genes, i.e.,* CSF3, CXCL10, FOS, HRAS, IL12A, IL1A, IRAK1, IRAK4, JUN, LTA, LY96, NFKB1, NFKB2, NFKBIA, NR2C2, RIPK2, SARM1, SIGIRR, TAB1, TLR2, TLR8,* and *UBE2N,* was observed HPBMs inoculated with *S. aureus* from men and women (*p* < 0.05) (Fig. 6B).

#### Comparison of the gene expressions of HPBMs of women and those of women treated with DHT

Among the 84 genes analysed, a statistically significant upregulation of 19 genes, i.e.,* BTK, CD80, EIF2AK2, IL12A, IL1A, IL1B, IL6, IRAK1, IRAK4, IRF3, JUN, LTA, NFKB2, NFKBIA, NR2C2, TLR2, TLR3, TLR8,* and *TNF,* was observed in the women HPBMs inoculated with *S. aureus* and pre-treated with DHT when compared to HPBMs from women (*p* < 0.05) (Fig. 6C).

## Discussion

The results of the present study provide evidence in support of the inflammatory effects of DHT in innate immunity. Using MPMs from male sham, male OQX, and female mice, and HPBMs from men and women healthy donors, we have shown that MPMs from male and HPBMs from men have higher expression and production of inflammatory cytokines than those from females and women respectively. This response is related to the action of the hormone testosterone. Supporting our hypothesis, a group of males castrated (orchiectomised-OQX) displayed lower concentrations of TNF-α, IL-1α, and IL-8 when compared with Sham males. When comparing sham males and females, sham males displayed higher concentrations of TNF-α, IL-1α, IL-6, and IL-8 and lower concentrations of IL-10. Similar results were observed with HPBMs; men displayed higher concentrations of TNF-α, IL-6, IL-12, and GM-CSF and lower concentrations of IL-10 when compared with women. A study in humans by Schröder et al. [31] showed that men display higher concentrations of TNF and IL-6 and women produce higher levels of anti-inflammatory cytokines and IL-10. This cytokine, as has been documented, plays an important beneficial role in suppressing inflammatory responses and improving the inflammatory profile in severe infections [32, 33]. A study by Von Aulock et al. [34] showed that the blood of men produced significantly more TNF-α, IL-1β, IL-6, and IL-8 than that of women in response to a high concentration of lipopolysaccharides (LPS) or lipoteichoic acid (LTA), corroborating with our results.

Our study showed that hormonal treatment with DHT increased the production of TNF-α, IL-1α, IL-6, and IL-8 and decreased the production of IL-10 in OQX ​​males. In corroborating these findings, HPBMs from women pre-treated with DHT displayed higher concentrations of TNF, IL-12, and GM-CSF and a lower concentration of IL-10. As reported, Th2 responses are usually activated and high levels of interleukins IL-4, IL-5, and IL-10 are usually produced in women in response to infections [31, 35]. Conversely, men predominantly display Th1 responses and overproduce TNF-α, IL-1β, IL-2, IL-6, and IL-8, which in turn is often associated with poor outcomes, such as septicaemia and bacteremia [36, 37]. Matalka et al. [38] observed that IL-10 levels were associated with the presence of oestrogen in women and were a protective factor. Since the concentration of 17-β estradiol (E2) increased, reaching concentrations corresponding to those found during pregnancy, IL-10 levels increased significantly. IL-10 is also released into the circulation during human sepsis and controls the production of TNF, IL-1, and IL-8 in vitro [36]. In this case, it is an advantage for the females.

Another salient finding of this study is that the MPMs of sham animals displayed lower concentrations of NO^−2^ and H_2_O_2_ when compared with OQX males. The orchiectomy can thus increase the release of free radicals by MPM-associated responses by *S. aureus*. In contrast, OQX males showed lower concentrations of these markers in cases of pre-treatment with DHT. When comparing genders, MPMs from sham males showed lower concentrations of NO^−2^ and H_2_O_2_ when compared with MPMs from females. We found similar results in HPBMs; men displayed lower concentrations of NO^−2^ and H_2_O_2_ when compared with women and, in relation to pre-treatment with DHT, the women's cells displayed lower concentrations of NO^−2^ and H_2_O_2._

Although testosterone has pro-oxidant properties, as shown by Azevedo et al. [37], this hormone inhibited the free radical-mediated response in our study. This effect of steroid hormones is, in part performed through the transcriptional regulation of certain enzymes involved in free radical metabolism [38]. Androgens are also known to have a role in inhibiting the activity of the NADPH enzyme complex, an important system as a microbicidal, free radical generating mechanism [37].

A study by Scotland et al. [39] found that the antibacterial activity of intracellular NADPH oxidase was significantly elevated in resident peritoneal macrophages in females when compared to males. Females should respond better to infection and show greater control of inflammatory mediators, such as free radicals. A study by Azevedo et al. [37] found that males produced lower levels of antioxidant enzymes in resident intraperitoneal macrophages when compared with females. Our hypothesis is that such an antioxidant response is harmful in the defence against *S. aureus* infections. Aerobic bacteria such as *S. aureus* develop antioxidant enzymes that inhibit free radical production by macrophages and prevent bacterial clearance and efficient defence responses of the immune system. *S. aureus* uses several strategies for its survival and proliferation in the host organism, such as complement opsonisation, neutralising phagocytosis, and inhibiting both humoral and cellular immune responses. Even the catalase enzyme produced by *S. aureus* converts H_2_O_2_, which would have a toxic effect on the bacteria, into oxygen and water [40]. In this case, both the dose and the inflammation model influenced the release of these markers [41–43]. A study by Xiao et al. [26] found that treatment with testosterone reduced the concentration of H_2_O_2_ by modulating the testosterone AR receptor in animal cardiac cells. Damage reduction was testosterone dose-dependent via the NF-κB pathway.

Considering the NF-κB pathway, our study did not find significant differences in TLR2 and NF-κB expression between MPMs from sham and OQX males infected with *S. aureus*. However, pre-treatment with DHT resulted in an increase in TLR-2 and NF-κB expression in MPMs from OQX males. One hypothesis for these findings is that the exogenous hormone may act through different pathways compared to the biological hormone. It is known that androgens can act via both the cellular and/or nuclear hormone receptor [44, 45] and directly on specific membrane-binding sites (SMBS). Testosterone, particularly exogenous testosterone, may have a non-genomic action via SMBS, an action not mediated by classical AR, but rather through the activation of membrane kinase signalling via the ERK and PK pathways, resulting in a series of biological activities dependent on this pathway, including expression of NF-κB, RelA (p65), Caspase-3, serine threonine kinase (Akt), extracellular signal-regulated kinase (ERK) 1/2, Bcl-2 and Bcl-xL, and protein kinases (PK) [20, 46].

In addition, our study showed that MPMs from sham males display higher expression levels of TLR2 and NF-κB when compared to those of females. In this case, the strong expression of these receptors provides a more uncontrolled response to *S. aureus* infection. An imbalance of TLR2, NF-κB, and pro- and anti-inflammatory cytokines usually results in an exacerbated inflammatory response, multiple organ dysfunction syndrome, and septic shock in humans [47, 48]. The analysis of the response in MPMs from females pre-treated with DHT showed no significant differences in TLR2 and NF-κB gene expression. However, this response in females is likely a consequence of the protective role of oestrogen by modulating the expression of TLR2 and NF-κB and providing greater balance in the release of cytokines and free radicals [49, 50]. Other studies pre-treated monocytes with E2 in vitro for 24 h before stimulation with LPS and found that NF-κB activation was suppressed in macrophages [51, 52]. These findings suggest that E2 exerts anti-inflammatory effects by modulating the response of receptors and cytokines [53], which can partially account for the sex differences in severe infections [31]. A study by Souza et al. [24] found that E2 reduced the production of TNF-α and decreased the expression of TLR2 and NF-κB in macrophages from mice infected with *S. aureus*. In our results, the hormone DHT had the opposite effect. The stimuli of expression of CD80 and CD86 probably promotes the expressions of TLR2 and transcription factors and DHT acts by increasing the expression of TLR2 and the transcription factors IRAK1, IRAK4, IKKβ and NFκB. Several extracellular stimuli, such as pro-inflammatory cytokines (IL-1, interleukin 1; TNF, tumor necrosis factor), cause phosphorylation (P) of IkB, which can be mediated by IkB kinase (IKK) and the TAB1 protein, known to mediate several intracellular signaling pathways, such as those induced by interleukin-1. Phosphorylation of IkB releases NF-kB which can act on a target gene in the nucleus, while IkB is degraded. However, there is a limitation in our trials to conclude this claim. New assays are needed to directly quantify the activation of the NF-kB pathway.

**Fig. 7:**
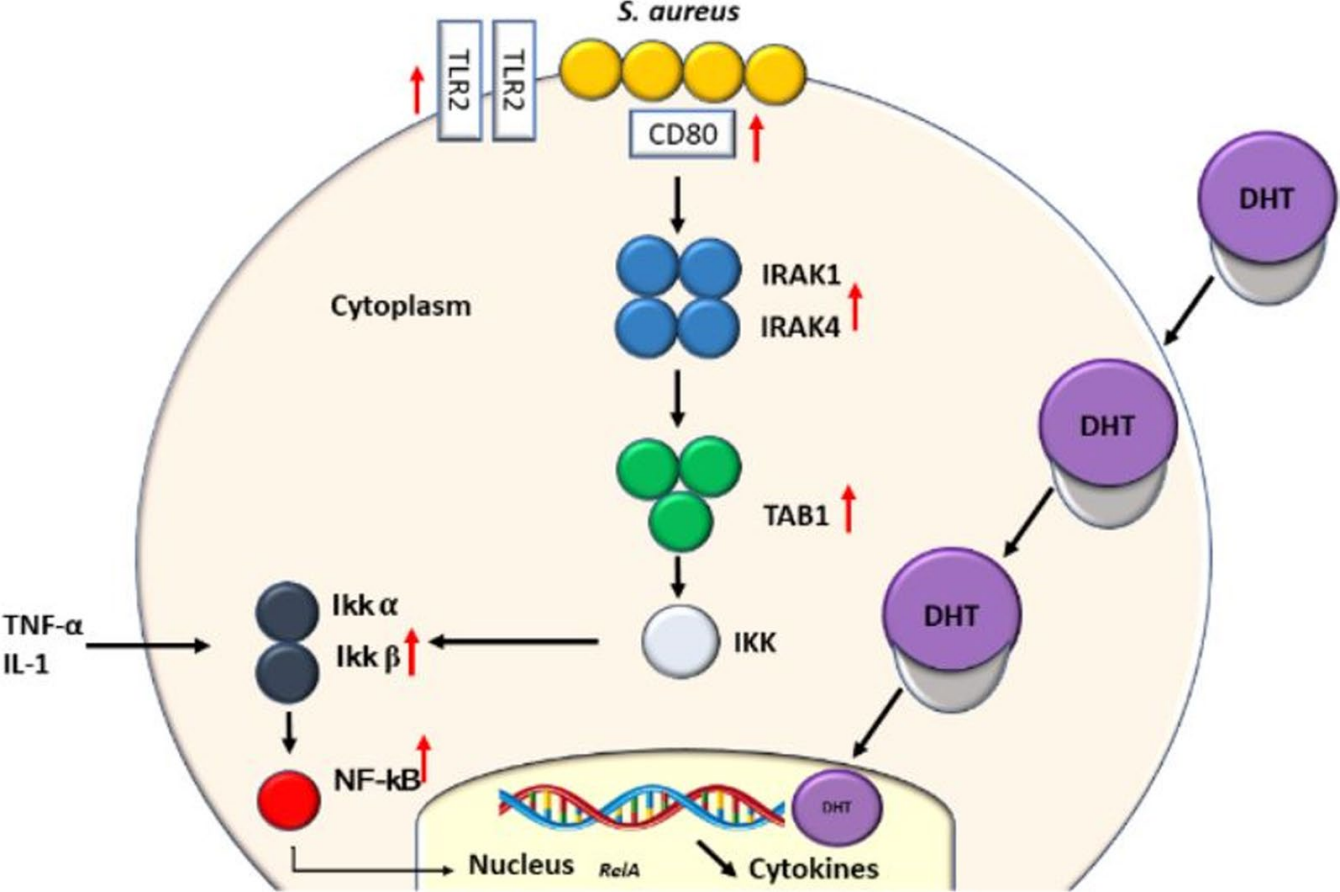
Effect of DHT on the immune response induced by *S. aureus*. DHT acts by increasing the expression of TLR2 and that of transcription factors IRAK1, IRAK4, IKKβ, and NFκB (represented by red arrows). Several extracellular stimuli, such as pro-inflammatory cytokines (IL-1, interleukin 1; TNF, tumour necrosis factor), cause IkB phosphorylation (P), which can be mediated by IkB kinase (IKK) and mediated by the TAB1 protein, known to mediate several intracellular signaling pathways, such as those induced by interleukin-1. The IkB phosphorylation releases NFκB which acts on a target gene in the nucleus, whereas IkB is degraded

CD80/CD86 signalling contributes to the pro-inflammatory response of *S. aureus*. This pathway is associated with cytokine release and macrophages activation [54]. T cells are activated through the interaction of peptides presented by the major histocompatibility complex (MHC), which interact with the T cell receptor complex, and lead to the initiation of several signalling cascades. This signalling complex is also induced through several co-receptors and their respective ligands. The CD28 T cell co-receptor interacts with CD80 (B7-1) and CD86 (B7-2) that are expressed on activated antigen-presenting cells in response to pathogens, such as *S. aureus*, which leads to the induction of several signalling pathways, such as those controlled by NF-κB, MAPK, PI3K, and AKT [55, 56]. In a study by Parker [54], CD80 and CD86-deficient mice displayed significant reductions in several pro-inflammatory cytokines and significantly improved survival rates in a murine model of pneumonia. This need for CD80 and CD86 in the pro-inflammatory response to *S. aureus* was evident early in the infection and in vitro using bone-marrow-derived macrophages. The work supported the hypothesis that much of the morbidity and mortality associated with *S. aureus* infection is the result of excessive production of cytokines. This brings us to the idea that DHT stimulates a more robust response of inflammatory cytokines, promoting a more intense response to *S. aureus* infection. Our results showed higher expression of genes such as *CD80*, *TNF*, *IL1A*, and *IL6* in HPBMs inoculated with *S. aureus* compared to in HPBMs inoculated with sterile saline (control). In addition to this response, we observed higher expressions of IL12A and TLR2 in HPBMs from men inoculated with *S. aureus* when compared to HPBMs from women. Corroborating our results, HPBMs of men displayed higher expression of TLR2 when compared with women. Our study also showed genes associated with upregulation in HPBMs inoculated with *S. aureus* from women treated with DHT compared to in HPBMs from women. We observed that DHT canupregulate genes that are involved in monocyte functions, such as the BTK gene [57], in addition to genes that encode proteins that phosphorylate the NF-κB pathway, such as: *CD80, TNF, IL12A, IL1A, IL1B, IL6, NFKB2, NFKBIA*, and *TLR*2. *S. aureus* can activate monocytes, whose activation is necessary for the initiation and modulation of the immune response, mainly through the transcription of genes related to the NF-κB pathway, consequently leading to the production/secretion of inflammatory pathway mediators and pro-inflammatory [58, 59] cytokines, such as TNF-α, IL-12, and IL6. Regardless of the stimulus, there seems to be a participation of ROS (oxidative stress) and an increase in intracellular calcium for the activation of NF-kB [60]. In a study by Yamamoto et al. [61] using cell culture, it was confirmed that LPS induced expression of the co-stimulatory molecules CD80 and CD86 macrophages. NF-kB is a master regulator of immunity and inflammation, and controls adhesion molecules, chemokines, growth factors, inflammatory enzymes, and pro-inflammatory. NF-kB is a heterodimer consisting of two subunits: p65 (also called RelA) and p50 [62, 63]. In the literature, the term authentic NF-kB designates the *p50/RelA* combination. In addition to these, other subunits have been described, such as *c-Rel, RelB*, and *p52*, and it is likely that different types of combinations are capable of activating different genes or blocking the transcription of *p50/RelA* [64]. In our results, we observed higher expression of *RelA* in HPBMs inoculated with *S. aureus* when compared to in those of the control. As given above, this study proposes that DHT stimulates the NF-κB pathway in the monocyte immune response induced by *S. aureus*, as outlined in Fig. 7.

### Perspectives and significance

The data show that females produce lower (potentially harmful) inflammatory cytokines, but produce more products to increase anti-inflammatory cytokines (IL-10). Although our model of study involved monocytes, we emphasize that these cells are applicable to what is seen in all diseases for sex differences, not just those associated with *S. aureus* infection. One of the most interesting results of our study is the data showing that TLR-2 increases with testosterone in men but not in women. This indicates that testosterone enhances pathogen recognition and is, in part the mechanism for the increase in dysregulated inflammation. Certainly, a protective response in HPBMs of women compared to men, and clearly show that the differences are related to testosterone. Thus, further studies are required to better understand these mechanisms of action of DHT in this inflammatory response pathway. We intend to analyze the profiles of other cells of the immune system in treatment with DHT in the future. It is expected that a better understanding of the role of testosterone in the body’s defense response against microorganisms may propose strategies for an effective, safe, and useful treatment. In addition, it may contribute to actions in promoting the population’s health, resulting in the reduction of costs with treatments, hospitalizations, complications, and improving the quality of life of people with this condition.

## Data Availability

The data sets used and/or analysed during the current study are available from the corresponding author on reasonable request.

## Abbreviations

BHI: Brain heart infusion
CAAE: Certificado de Apresentação para Apreciação Ética (Certificate of Submission for Ethical Consideration)
CFU: Colony Forming Units
DHT: Dihydrotestosterone
E2: 17β-Oestradiol
ELISA: Enzyme-linked immunosorbent assay
FBS: Foetal bovine serum
GAPDH: Glyceraldehyde 3-phosphate dehydrogenase
GM-CSF: Granulocyte and macrophage stimulating factor
HPBMs: Human peripheral blood monocytes
H_2_O_2_: Hydrogen peroxide
IFN-γ: Interferon-gamma
IL-1α: Interleukin 1 alpha
IL-6: Interleukin 6
IL-8: Interleukin 8
IL-10: Interleukin 10
IL-12: Interleukin 12
JNK: C-Jun N-terminal kinase
MAPK: Mitogen-activated protein kinase
MHC: Major histocompatibility complex
MPMs: Mouse peritoneal macrophages
NF-κB: Nuclear factor kappa B
NFKBIA: NFKB inhibitor alpha
NO^−^^2^: Total nitrites
OQX: Orchiectomised males
P38: Mitogen-activated protein kinases
ROS: Reactive oxygen species
RPMI: Roswell Park Memorial Institute (culture medium)
RT-qPCR: Reverse transcription quantitative PCR
SAPK: Interacting protein
SHAM: Males submitted to simulated surgery (sham surgery)
SPF: Specific-pathogen-free
TLR2: Toll-like receptor 2
TNF-α: Tumour necrosis factor alpha
